# HDACi Valproic Acid (VPA) and Suberoylanilide Hydroxamic Acid (SAHA) Delay but Fail to Protect against Warm Hepatic Ischemia-Reperfusion Injury

**DOI:** 10.1371/journal.pone.0161233

**Published:** 2016-08-11

**Authors:** Dietrich A. Ruess, Moriz Probst, Goran Marjanovic, Uwe A. Wittel, Ulrich T. Hopt, Tobias Keck, Dirk Bausch

**Affiliations:** 1 Department of Surgery, University Hospital Freiburg, Freiburg, Germany; 2 Department of Surgery, University Hospital Schleswig-Holstein, Campus Lübeck, Lübeck, Germany; Vrije Universiteit Brussel, BELGIUM

## Abstract

**Background:**

Histone deacetylases (HDAC) catalyze N-terminal deacetylation of lysine-residues on histones and multiple nuclear and cytoplasmic proteins. In various animal models, such as trauma/hemorrhagic shock, ischemic stroke or myocardial infarction, HDAC inhibitor (HDACi) application is cyto- and organoprotective and promotes survival. HDACi reduce stress signaling, cell death and inflammation. Hepatic ischemia-reperfusion (I/R) injury during major liver resection or transplantation increases morbidity and mortality. Assuming protective properties, the aim of this study was to investigate the effect of the HDACi VPA and SAHA on warm hepatic I/R.

**Material and Methods:**

Male Wistar-Kyoto rats (age: 6–8 weeks) were randomized to VPA, SAHA, vehicle control (pre-) treatment or sham-groups and underwent partial no-flow liver ischemia for 90 minutes with subsequent reperfusion for 6, 12, 24 and 60 hours. Injury and regeneration was quantified by serum AST and ALT levels, by macroscopic aspect and (immuno-) histology. HDACi treatment efficiency, impact on MAPK/SAPK-activation and Hippo-YAP signaling was determined by Western blot.

**Results:**

Treatment with HDACi significantly enhanced hyperacetylation of Histone H3-K9 during I/R, indicative of adequate treatment efficiency. Liver injury, as measured by macroscopic aspect, serum transaminases and histology, was delayed, but not alleviated in VPA and SAHA treated animals. Importantly, tissue destruction was significantly more pronounced with VPA. SAPK-activation (p38 and JNK) was reduced by VPA and SAHA in the early (6h) reperfusion phase, but augmented later on (JNK, 24h). Regeneration appeared enhanced in SAHA and VPA treated animals and was dependent on Hippo-YAP signaling.

**Conclusions:**

VPA and SAHA delay warm hepatic I/R injury at least in part through modulation of SAPK-activation. However, these HDACi fail to exert organoprotective effects, in this setting. For VPA, belated damage is even aggravated.

## Introduction

Histone deacetylases (HDACs) are a group of enzymes involved in epigenetic regulation and chromatin remodeling. Partly as catalytic subunits of large protein complexes, HDACs are also capable of removing acetyl groups from lysine residues of different nuclear and cytoplasmic proteins, thus altering intracellular signaling [1].

The family of HDACs consists of four classes. Commonly used HDACi target multiple HDACs and more than one class. This is also true for valproic acid (VPA, inhibits class I: HDAC 1, 2, 3, 8 and class IIa: HDAC 4, 5, 7, 9) and SAHA (in addition to class I and class IIa inhibits class IIb: HDAC 6, 10 and class IV) [2].

HDACi may exert cyto- and organoprotective effects in liver, lungs and kidneys with beneficial impact on survival in rodent and swine models for polytrauma/hemorrhagic shock [3–7]. In further preclinical *in vivo* studies evaluating brain trauma [8], ischemic stroke [9,10] and various ischemia-reperfusion injuries (cerebral I/R [11], myocardial I/R [12,13], renal I/R [14], supracoeliac I/R [15]) HDACi provided neuroprotection, reduced infarct volumes/residua, and blunted I/R injury, respectively.

Pathophysiology of hepatic I/R is complex. Hepatocyte cell death mainly ensues by necrosis, but also by apoptosis [16–19]. Activation of all mitogen/stress activated protein kinases (MAPK/SAPK) ERK, JNK and p38 plays an important role in hepatic I/R injury. While extracellular signal-related kinases ERK1/2 seem to play a protective, pro-survival role [20–23], activation of c-Jun N-terminal kinases (JNK)- and p38-signaling is mainly deleterious [22,24–27].

The Hippo pathway is a potent regulator of organ size and tissue homeostasis [28]. It has substantial implications for hepatic regeneration [29,30]. Upon tissue insult/loss, Hippo signaling is switched off. Unphosphorylated YAP then translocates to the nucleus and exerts pro-proliferative and anti-apoptotic functions.

Hepatic I/R injury can occur during major liver resection with temporary vascular exclusion (Pringle maneuver) or during liver transplantation, where, next to organ rejection, I/R injury is a major risk factor. It substantially affects postoperative liver function, thus considerably increasing morbidity and mortality.

We hypothesized that (pre-) treatment with VPA and/or SAHA, feasible for organ donors or prior to major liver surgery in a clinical setting, might exert protective effects in warm hepatic ischemia-reperfusion injury in a preclinical rodent model. Recently, this was demonstrated for butyrate (inhibits class I and class IIa HDAC) [31]. We here chose to compare two clinically readily available pan-HDACi with (VPA) and without (SAHA) potential hepatotoxic properties. Additionally, we were interested in a potential impact of these agents on liver regeneration after I/R insult.

## Material and Methods

### Animals, treatment protocol and surgical procedure

**Fig 1:**
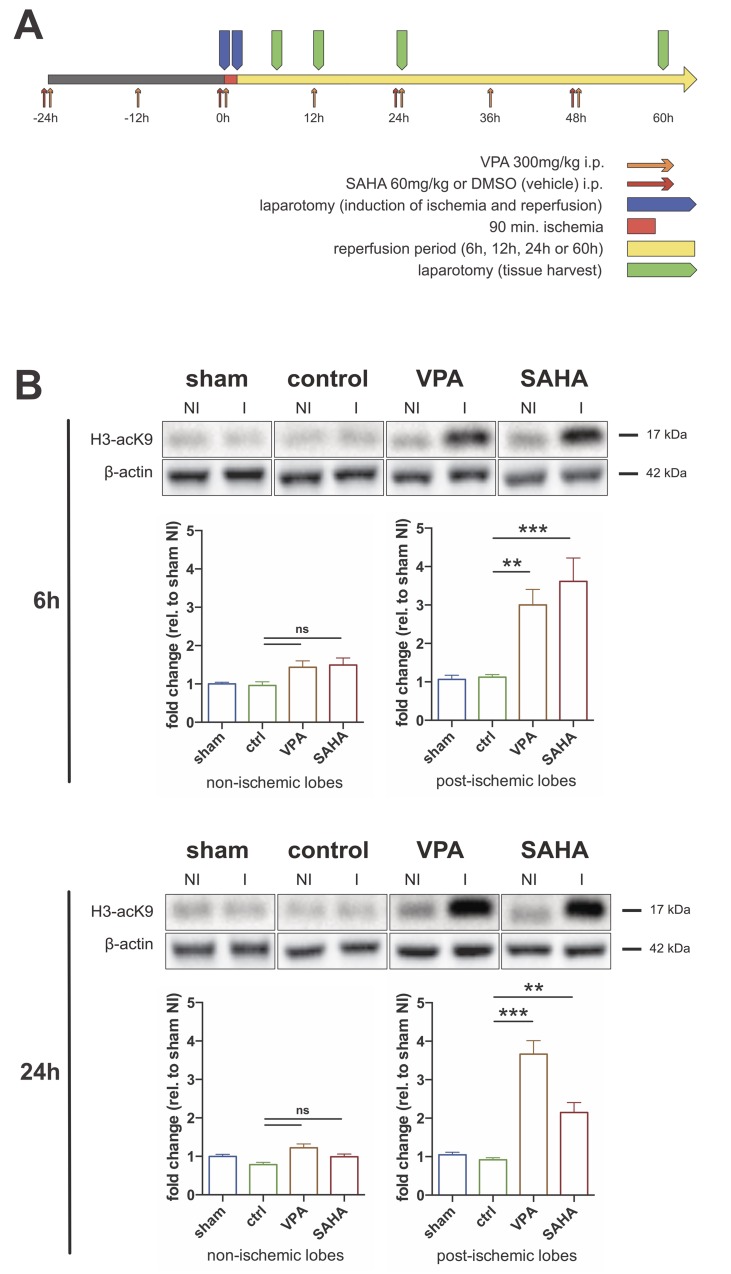
VPA and SAHA reinforce I/R-mediated hyperacetylation of Histone H3. **A)** Time-schedule of the performed experiments. VPA: valproic acid; SAHA: suberoylanilide hydroxamic acid; DMSO: vehicle dimethylsulfoxide. **B)** Western blot for acetylated Lysine 9 of Histone H3. β-actin served as loading control. Representative blots for 6h and 24h of reperfusion are shown. Bar graphs depict quantification of at least four experiments. NI: non-ischemic right lobes; I: ischemia-reperfusion left lobes. ***: p<0.001; **: p<0.01.

Male *Wistar-Kyoto* rats (weight: 200-250g; age: 6–8 weeks) were purchased from Charles River and maintained in the animal research facility at University Hospital Freiburg. Treatment with 300mg/kg Valproic Acid (VPA: Orfiril**®**, Desitin, 100mg/ml) or 60mg/kg Suberoylanilide Hydroxamic Acid (SAHA: Vorinostat, Cayman Chemicals, 20mg/ml in Dimethylsulfoxide (DMSO)) was initiated by intraperitoneal administration 24h before onset of ischemia. In the further course injections were repeated every 12h or 24h for VPA or SAHA, respectively (**Fig 1A**) [3,9,32]. Control animals were vehicle (DMSO)-treated and injected in analogy to the SAHA-protocol.

Surgery for initiation of partial warm no-flow ischemia and subsequent reperfusion was performed according to the model described by Jaeschke and colleagues [33]: Under inhalation anesthesia with isoflurane the blood supply to the left lateral lobe and left part of the median lobe of the liver was occluded by clamping. Reperfusion was accomplished by removal of the clamp after 90 minutes. Sham-treated rats underwent the same procedure except for placing the clamp. Sacrifice and tissue harvest was performed 6, 12, 24 and 60 hours after reperfusion (**Fig 1A**). For histologic analysis horizontal slices (3-5mm) from the middle of each lobe (left lateral lobe and left part of median lobe (I/R) vs. right part of median lobe and superior right lateral lobe (no I/R) were fixed in 4% formalin. Remaining tissue was cut into small pieces, pooled for I/R vs. no I/R lobes and snap-frozen in liquid nitrogen.

Experiments were approved by the regional commission for animal protection (Regierungspräsidium Freiburg; G-12/025). No animals utilized in this study became severely ill or died prior to the experimental endpoint. No animals received medical treatment or were humanely euthanized due to symptoms indicative of severe illness/moribundity.

### Macroscopic quantification

Macroscopic evaluation was performed on photographed sequential slices (3-4mm) of the left lateral lobe. Damaged and total areas of each single slice were determined using ImageJ software (National Institutes of Health, Bethesda, MD, USA). Integration over all slices yielded damaged and total volumes, which were set in relation to one another.

### Serum analysis

Whole blood was collected in Serum monovettes (Sarstedt), allowed to clot, and centrifuged at 4°C, 1500g for 10 minutes. Serum was frozen at -80°C until further analysis. Levels of aspartate transaminase (AST), alanine transaminase (ALT), and Valproic Acid (VPA) were determined by the *Institute for Clinical Chemistry and Laboratory Medicine*, *University Hospital Freiburg*, *Germany*.

### Histology and immunohistochemistry

Formalin-fixed tissue samples were embedded in paraffin, cut to 3–5μm sections, mounted on slides, baked at 55°C for 45 minutes and stained with hematoxylin and eosin (H/E) or stored at room temperature.

For Ki67 and (p-)YAP immunohistochemistry hydrated slides were boiled in target retrieval solution pH 9 (Dako, S2367). Ki67 clone MIB-5 (Dako, M7248) was used in a dilution of 1:50; YAP (#4912) and p-YAP (#4911) antibodies (Cell Signaling Technology) in dilutions of 1:400 and 1:500 respectively. Biotinylated secondary antibody (Vector Laboratories, BA2000), 1:200, was applied and signal was enhanced by Vectastain ABC Kit followed by development with AEC Substrate Kit (Vector Laboratories, PK4000 and SK4200). Slides were counterstained with Mayer’s Hemalaun, dehydrated, cleared and mounted.

Stained tissue sections were analyzed on a BXiS microscope (Olympus, Inc.). At least 20 high power fields (x100) per IHC-section were subjected to counting. For H/E-staining one whole section per animal was quantified (magnification: x40 and x100). Damaged areas as determined by sinusoidal jamming, loss of eosinophilia, cell vacuolization, leucocyte infiltration, loss of tissue-architecture and scar formation were set into relation with the total area of the whole section.

Identification of the predominant form of cell death was accomplished by (H/E-) morphological criteria such as cell shrinkage, chromatin condensation/margination and apoptotic bodies for apoptosis, and vacuolization, cell disruption/loss of architecture, karyorrhexis/karyolysis and loss of nuclei for necrosis.

### SDS-PAGE and Western blot

Snap-frozen liver tissue samples were homogenized and lysed in RIPA-buffer. After homogenization samples were stored on ice for 15 minutes followed by centrifugation with 16000g for 20 minutes at 4°C. The supernatant was collected carefully with a fine syringe, leaving an occasional lipid layer and the pellet behind. Protein quantification was performed with Pierce BCA Protein assay kit (ThermoFisherScientific). Adjusted Laemmli-samples were boiled at 95°C for 5 minutes and loaded on precast 12% Mini-PROTEAN**®** TGX Stain-free gels (Bio-Rad), 30μg/lane. After electrophoresis the Stain-free gels were subjected to image-analysis for loading control on the Biorad ChemiDoc**™** system and subsequently blotted to a 0.45μm pore-size Nitrocellulose membrane. The following antibodies were used: Primary antibodies: Acetyl-Histone H3 (Lys9) (07–352) from Millipore, ERK1/2 (Ab17942), p-ERK1/2 (Ab50011), p38 (Ab31828) and p-p38 (Ab35227) from Abcam, JNK (#9258), p-JNK (#4668), YAP (#4912), p-YAP (#4911) from Cell Signaling Technology. Secondary antibodies: HRP-anti-rabbit (AK9340V) by Amersham/GE, HRP-anti-rabbit (32460) and HRP-anti-mouse (31430) from Pierce/ThermoFisherScientific. Total protein amount was additionally confirmed via β-actin (A5316, Sigma).

Chemiluminescent detection was carried out with SuperSignal West Pico substrate (34080, ThermoFisherScientific) and the Biorad ChemiDoc**™** system. Densitometric quantification was accomplished via Biorad ImageLab**™** software. For acetyl-Histone H3 (Lys9) expression was normalized to total protein taking advantage of the stain-free gels. Phosphorylated MAP-kinases and YAP were set in relation to total expression of the corresponding proteins.

### Statistical analysis

Statistical analysis was performed using GraphPad Prism, GraphPad Software, Inc. Data are displayed as mean ± SEM. Comparisons among the different treatment groups or reperfusion periods were accomplished by analysis of variance (ANOVA) and post-hoc Tukey’s or Dunnett’s correction for multiple comparisons. P<0.05 was considered significant.

## Results

Rats were randomly assigned to one of the 16 experimental groups (n = 3–6 each). **Fig 1A** gives an overview of the experimental protocol and time frame.

### VPA and SAHA reinforce I/R-mediated hyperacetylation of Histone H3

HDACi treatment effect was monitored by Western blot for acetylated Lysine 9 of Histone H3 (H3-acK9) of whole tissue liver lysates. Significant hyperacetylation by VPA and SAHA compared to control-treated animals was only observed in postischemic I/R-lobes at all time points (6h and 24h are shown in **Fig 1B**).

Additionally, for VPA serum concentration in VPA treated I/R animals showed adequate levels for HDAC-inhibition. Values exceeded the required threshold concentration of ~86mg/l [3] for as long as about five hours post-injection (**S1 Fig**). In vitro, histone-acetylation peaks at four hours after treatment and lasts more than 36 hours [34]. VPA-injections were repeated every 12 hours in our protocol.

### VPA- and SAHA-treatment delays I/R-damage but fails to exert protective properties

**Fig 2:**
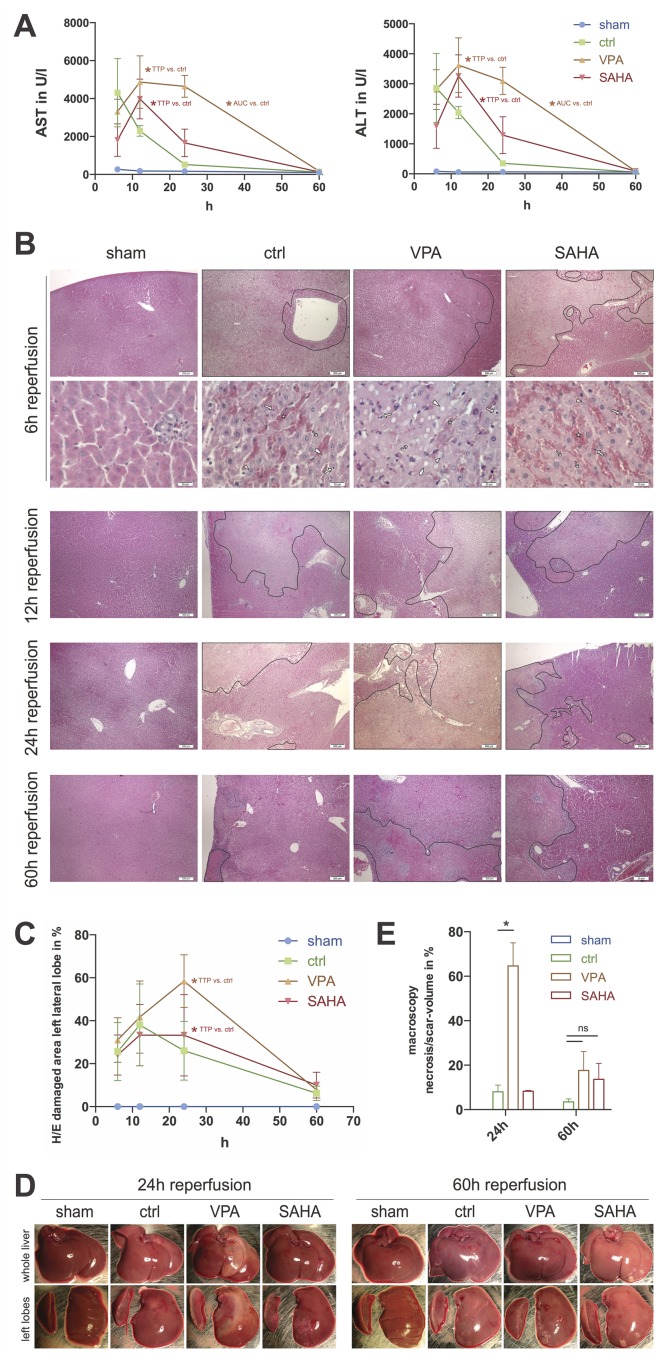
VPA- and SAHA-treatment delays I/R-damage but fails to exert protective properties. **A)** Serum levels of aspartate (AST) and alanine (ALT) aminotransferases at different time points. **B)** Representative H/E micrographs of left lateral liver lobe sections from animals treated as indicated. On the images with lower magnification (x40, scale bars 200μm) damaged areas are encircled. The images with higher magnification (lower row of 6h reperfusion; x400, scale bars 20μm) depict sinusoidal jamming (star) and signs of hepatocyte cell death like karyopyknosis (thick arrow), karyorrhexis (arrowhead), karyolysis (fine arrow) and loss of nuclei (raindrop) for all treatment options as indicated. **C)** Histological quantification of tissue damage. One whole representative horizontal section per animal was analyzed. Percentage of damaged area (includes: sinusoidal jamming, cell death/scar and inflammatory infiltrates) is given. **D)** Representative photographs of whole liver (top row), and dissected left lateral and left part of median liver lobe (bottom row) of animals subjected to the indicated treatments and sacrificed at indicated timepoints. **E)** Quantification of I/R-damage by macroscopic aspect of the left lateral liver lobe. Integrated volume of necrosis/scar was set in relation to the calculated total volume of the lobe. *: p<0.05. TTP: time to peak; AUC: area under the curve.

In animals subjected to 90 minutes of partial no-flow ischemia and subsequent reperfusion, serum levels of ALT and AST peaked 6±0h (mean±SEM) after reperfusion for the vehicle control treated group. In serum of rats treated with VPA or SAHA levels of AST and ALT were lower at 6h, compared to controls. Here, the peaks were detected significantly later, at 20±4h or 15±3h of reperfusion for AST (p<0.05) and 16±4h or 15±3h of reperfusion for ALT (p<0.05), respectively (**Fig 2A**).

Quantification of H/E-histology yielded similar results. For vehicle control treated animals the relative amount of damaged areas peaked at 7.2±1.2h of reperfusion. SAHA treatment delayed this peak to 19.5±4.5h (p<0.01). VPA treated livers even more lagged behind, demonstrating maximum histological damage at 24±0h of reperfusion (p<0.01) (**Fig 2B and 2C**).

However, regarding integrated damage, for SAHA treated animals no significant benefit compared to controls was detectable. The area under the curve (AUC) of transaminases and H/E-histology as well as macroscopic aspect and quantification were similar.

Importantly, in the case of VPA treatment, I/R-damage was not only delayed but also aggravated compared to vehicle controls, as measured by the area under the curve (AUC) of the serum transaminase levels (AST: 168±28 vs. 46±10, p<0.05; ALT: 117±21 vs. 32±8, p<0.05) (**Fig 2A**). AUC for H/E damage quantification was not significantly different between VPA and control groups, nevertheless macroscopic aspect at tissue harvest did display enhanced damage: I/R lobes of VPA treated animals with 24h reperfusion appeared more pale and with more areas suggestive of necrosis and scar, compared to the other groups and time periods (**Fig 2D**). Volumetric quantification of macroscopic tissue damage of the left lateral lobes confirmed this notion. At 24h reperfusion the proportion of damaged tissue was significantly higher in VPA treated livers, compared to vehicle controls (64±10% vs. 8±3%, p<0.05). At 60h reperfusion a difference between VPA and controls remained but was not significant (**Fig 2D and 2E**).

### VPA and SAHA modulate I/R-dependent activation of p38- and JNK-signaling

Activation of all mitogen/stress activated protein kinases (MAPK/SAPK) extracellular-signal regulated kinases ERK1/2, c-Jun N-terminal kinases JNK, and p38 has been shown to play an important role in hepatic I/R injury. ERK1/2 seems to play a protective, pro-survival role, activation of JNK and p38 is mainly deleterious. In I/R models of different organs other than liver HDACi have been able to beneficially modulate MAPK/SAPK activation responses. To evaluate a potential impact of HDAC inhibition by VPA and SAHA on activation of the MAPKs/SAPKs ERK1/2, JNK and p38 in our partial warm liver I/R model we performed Western blot analyses.

**Fig 3:**
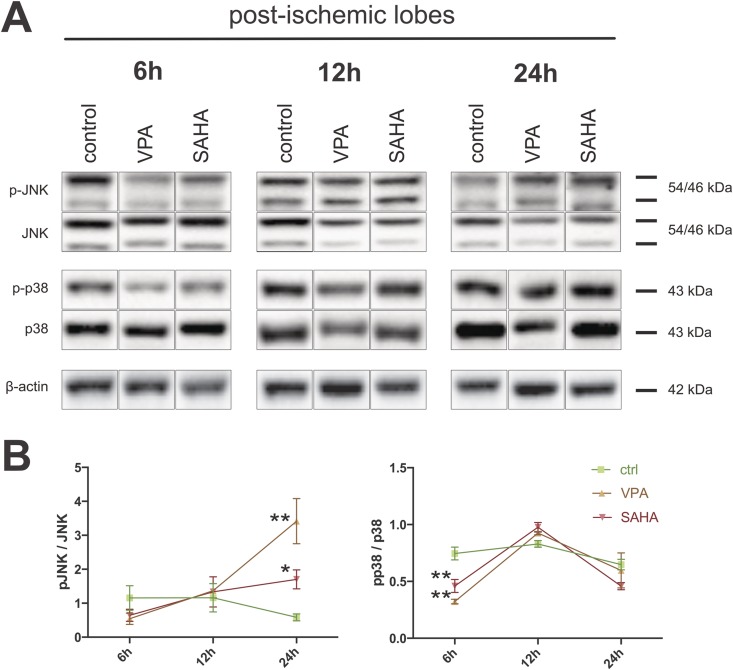
VPA and SAHA modulate I/R-dependent activation of p38- and JNK-signaling. **A)** Representative Western blots for (phospho-)JNK (T183/T185) and (phospho-)p38 (T180/Y182). β-actin served as loading control. **B)** Densitometric blot-quantifications for indicated treatment groups and reperfusion periods of at least three experiments. NI: non-ischemic right lobes; I: post-ischemic I/R left lobes. **: p<0.01; *: p<0.05.

Both stress activated protein kinases p38 and JNK were phosphorylated and activated early on during reperfusion. For p38 this was the case in both left *and* right lobes. JNK phosphorylation seemed to be restricted to the post-ischemic left lobes (data not shown). More importantly, early activation of p38 was significantly dampened by VPA (p<0.01) and SAHA treatment (p<0.01) at 6h reperfusion (**Fig 3A and 3B**). In addition, JNK phosphorylation was more pronounced in VPA (p<0.01) and SAHA treated (p<0.05) post-ischemic livers at 24h reperfusion. (**Fig 3A and 3B**). For ERK1/2 we did not detect significant differences in activation levels (data not shown).

Together, these results confirm activation of SAPK/MAPK signaling by partial warm liver I/R. Further, they indicate a potential of HDACi VPA and SAHA to attenuate SAP-kinase (p38 and JNK) -activation in the early reperfusion phase (6h). However, this effect is lost at 12h and for JNK even reversed in the further course (24h).

### Impact of HDAC-inhibition on regeneration

In order to investigate the role of HDAC inhibition by VPA and SAHA on regeneration after I/R damage we performed immunohistochemistry for the proliferation marker Ki67 and Western blot analyses as well as immunohistochemistry for the Hippo signaling downstream effector (phospho-) YAP.

**Fig 4:**
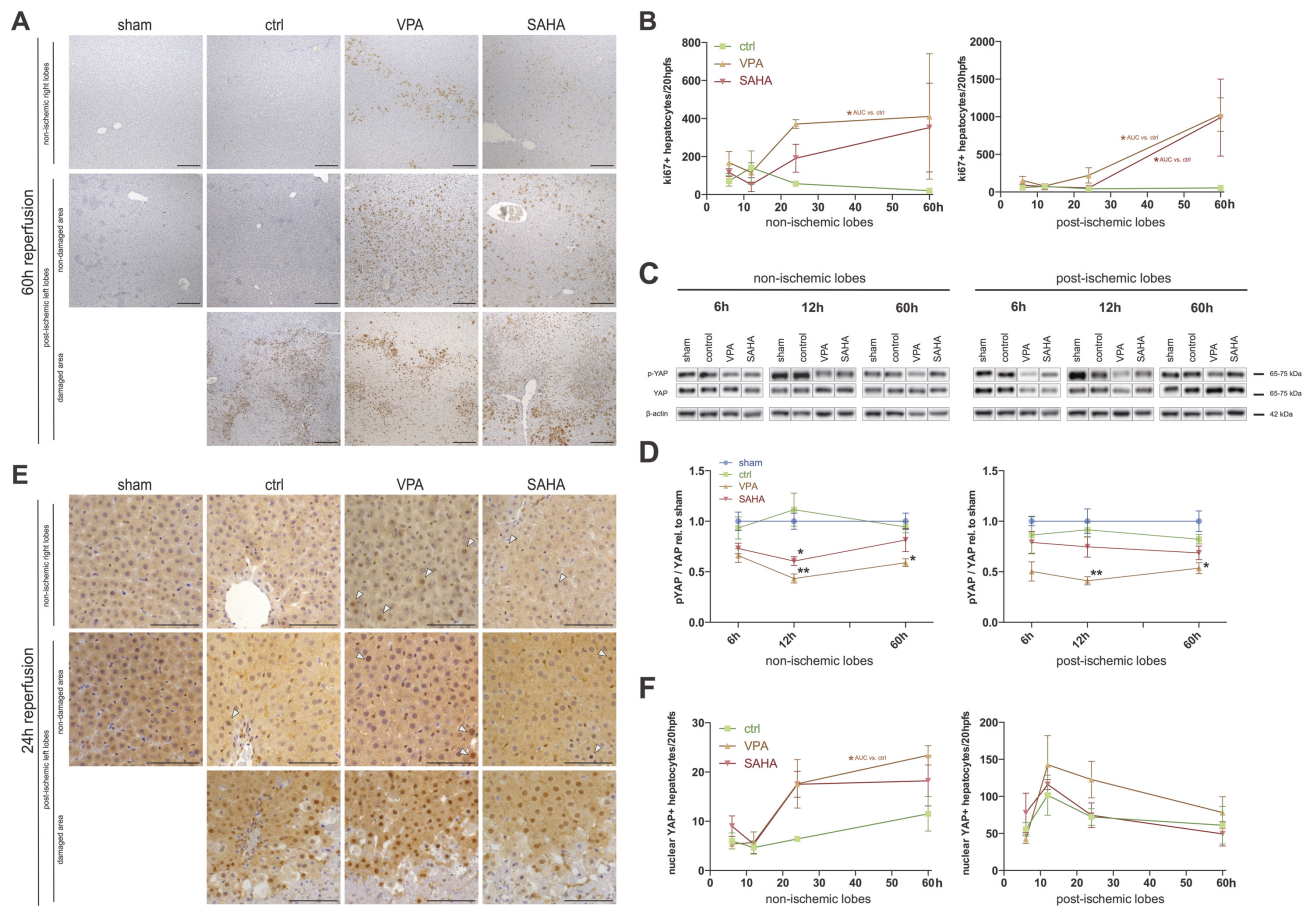
Impact of HDAC-inhibition on regeneration. **A)** Ki67 immunohistochemistry. Representative micrographs of non-damaged and damaged areas in sections of post-ischemic left liver lobes and of non-ischemic control lobes from animals treated as indicated. Scale bars: 200μm. **B)** Quantification of Ki67-positive hepatocytes in I/R unaffected areas. At least 20 HPFs of areas without massive inflammatory infiltrates and scar tissue were counted per slide. **C)** Representative Western blots with (phospho-)YAP (S127) antibodies and **D)** Graphs of densitometric quantifications of at least three experiments. Values were set in relation to the samples of sham treated animals. **E)** YAP immunohistochemistry. Representative micrographs of non-damaged and damaged areas in sections of post-ischemic left liver lobes and of non-ischemic right control lobes from animals treated as indicated. White arrows indicate nuclear staining in the images for non-ischemic lobes and the non-damaged areas of post-ischemic lobes. Arrows were omitted in images for damaged areas of post-ischemic lobes. Scale bars: 100μm. **F)** Quantification of nuclear YAP-positive hepatocytes. At least 20 HPFs were counted per slide. NI: non-ischemic right lobes; I: post-ischemic I/R left lobes. **: p<0.01; *: p<0.05. AUC: area under the curve.

Regenerative hepatocyte proliferation was strongly augmented in both parts of VPA treated livers (AUC NI: 19±7, p<0.05; AUC I: 27±7, p<0.05) and in post-ischemic lobes of SAHA treated livers (AUC: 20±9, p<0.05), compared to vehicle controls (AUC NI 2.3±0.6; AUC I 2.4±0.7) (**Fig 4A and 4B**). Quantification values for Ki67-positive proliferating hepatocytes were mostly similar in corresponding post-ischemic and non-ischemic lobes. Only in case of very high damage to the left lobes (especially with VPA treatment), compensatory proliferation in the right lobes was noticed. At 60h, regeneration did not yet take place in necrotic zones, but was pronounced at their borders to healthy tissue and in regions with favorable supply around greater vessels or close to the organ surface/peritoneum. However, slightly lower rates of Ki67 positive hepatocytes were also distributed diffusely in remaining unaffected tissue. (**Fig 4A and 4B**).

We could not observe significant dephosphorylation of YAP by mere I/R in the post-ischemic lobes of the vehicle control treated group, compared to sham procedure animals. However, with VPA treatment dephosphorylation of YAP in right *and* left liver lobes was detected from 12h (p<0.01) to 60h (p<0.05) of reperfusion. For SAHA, relative unphosphorylated YAP-levels were significantly lower only in non-ischemic lobes at 12h reperfusion (**Fig 4C and 4D**). Notably, total-YAP expression was downregulated under HDACi treatment in post-ischemic lobes and the early reperfusion-phase (6h and 12h), regaining baseline values until 60h (**Fig 4C**). Immunohistochemistry for YAP revealed very rare nuclear staining in livers of sham treated animals. With I/R damage this was increased strongly in post-ischemic parts especially at the borders of necrotic areas, but to a lesser extent also in the non-ischemic parts of the control cohort livers. In line with the Western blot analyses, VPA treatment significantly further augmented nuclear YAP localization in non-ischemic lobes (p<0.05) and showed a trend towards the same effect in post-ischemic lobes. For SAHA, a trend towards increased cell numbers with nuclear YAP, compared to vehicle control could be observed in the non-ischemic lobes (**Fig 4E and 4F**). Immunohistochemistry for p-YAP corroborated these findings (**S2 Fig**).

These results illustrate an enhanced regeneration response in SAHA and especially VPA treated animals. Hippo/YAP dependent signaling is involved in this response.

## Discussion

It has been previously shown that HDACi potently reduce cellular and organ damage, promote survival and attenuate inflammation in multiple animal models of polytrauma, hemorrhagic and septic shock, permanent ischemia and diverse ischemia-reperfusion injuries [3,4,6–8,10–15,31,35,36]. Partly, this effect has been attributed to modulation of intracellular signaling pathways such as the MAP kinase cascades [7,35–37].

In this study we addressed the question whether prophylactic therapy with VPA/SAHA may reduce hepatic I/R damage and impact the regeneration phase after injury. In the clinical setting this approach would be feasible before major liver resection or organ harvest for transplantation. We utilized an established rat model with partial no-flow ischemia for 90 minutes and subsequent reperfusion for 6–60 hours.

First, we detected an only very slight increase in acetylation status of histone H3 by I/R, which was markedly potentiated by administration of VPA and SAHA. Others have reported either I/R-mediated deacetylation of H3, rescued by the HDACi butyrate in a rat model [31], or hyperacetylation of H3 via loss of nuclear HDAC-activity by I/R in a murine model [38], both with 60 minutes partial no-flow ischemia. Species dependent effects, as well as the timing and magnitude of the initiating ischemic insult and pharmacologic treatment protocol may be responsible for these differences. The results of our study imply that in this specific setup neither I/R alone, nor the amount of VPA/SAHA applied, but only the combination of both leads to significantly elevated H3K9 acetylation in liver tissue lysates. However, since we did not see dominant changes of H3-acetylation in the non-ischemic right lobes of VPA and SAHA treated compared to control animals, we further cannot exclude a bias by abundant infiltrating immune cells as evident on H/E and ki67-stainings in the post-ischemic left lobes, which could be a cellular source for hyperacetylated H3K9.

In our experiments we then observed the predominant mode of cell death being oncotic necrosis. Varying degrees of apoptosis and necrosis have been shown in different hepatic ischemia/reperfusion protocols. In principle, the duration of the ischemia-period may influence the amount of ATP-depletion favoring either the energy dependent form of programmed cell death or necrosis. In addition, apoptosis can be detected rather early by e.g. cleavage of caspases 3/8, expression of proapoptotic proteins BAX/BAD and/or antiapoptotic proteins like Bcl-2. In contrast, necrosis becomes histologically apparent later, usually not before 3h of reperfusion [39]. However, our results are in line with those from *Jaeschke’s* group, who demonstrated for the identical rat I/R model with ischemia-periods of 45, 60 and 120 minutes that less than 2% of hepatocytes and sinusoidal epithelial cells undergo apoptosis, while the major part of damaged cells dies by necrosis [33]. It has been long known that TUNEL-staining can also mark DNA-strand breaks in necrotic cells [40]. This lack of specificity was confirmed in our setting, where TUNEL-staining was not helpful to discern necrotic and apoptotic cells (data not shown).

According to previous studies [33,41] the serum- and H/E histological peaks of damage in our vehicle control treated group emerged around 6h and 12h reperfusion, respectively. With VPA and SAHA treatment maximum serum ALT/AST levels and greatest microscopic damage were delayed. With SAHA the AUCs of serum-transaminases and of histological damage were not larger, but also not smaller than in controls. Nonetheless, for VPA treated animals the AUC for ALT/AST as well as histological and macroscopic damage exceeded those from vehicle injected rats. Although SAHA is metabolized in the liver, follows biliary excretion and is to be dose-adapted with liver disease, no hepatotoxic effects have been described. In contrast, clinical and experimental data indicate hepatotoxicity associated with VPA. It may present as rather chronic evolving liver failure with long-term treatment or may infrequently develop rapidly as potentially lethal Reye-like syndrome. Given the low incidence of VPA-associated liver failure, idiosyncratic toxicity and individual susceptibility play a major role [42]. Mechanistically, VPA and its metabolites can lead to mitochondrial dysfunction, oxidative stress, lipid peroxidation, microvesicular steatosis, and cell death [43–48]. To achieve an HDACi-effect with VPA, serum concentration necessarily needs to reach values approximately 3–4 fold of those needed for antiepileptic activities [3]. The rather high doses applied to our animals resulted in adequate, but not exuberant serum levels and are therefore required. Interestingly, we did not observe any obvious macroscopic or histologic liver damage in the non-ischemic lobes of VPA-treated animals. However, (delayed) maximum injury in the post-ischemic lobes was significantly potentiated compared to control animals. We assume a synergistic amplification of oxidative stress with I/R and cumulative VPA-injections.

Regarding the protective impact of VPA and SAHA in the early reperfusion phase (6h) we found an attenuation of SAP-kinase (p38 and JNK) activation. With longer reperfusion periods this effect was lost, and even reversed for JNK. Cell injury and death trigger the release of damage-associated molecular patterns (DAMPs), which interact with toll like receptors (TLR2/4) mainly on immune cells triggering an inflammatory response. Major players in TLR-signaling are NFκB and p38/JNK [49]. TLR expression in hepatocytes is upregulated in a NFκB-dependent manner implying acquirement of higher responsiveness to DAMPs in inflammatory conditions [50]. In addition to DAMPs, potent activators of SAPKs are stress-signals like cytokines, heat shock, (UV-) radiation, lipopolysaccharide and oxidative stress itself [27]. Several studies have ascribed deleterious impact on hepatic I/R injury to p38 [22,25] and JNK [22,24,26,51]. In response to activation by I/R, inflammatory mediators are produced and secreted, and cell death is promoted (p38: mainly apoptosis, JNK: apoptosis [27] or necrosis [51]). In rodent models for hemorrhagic and septic shock HDACi treatment attenuates SAPK activation and inflammation [7,37]. Importantly, the impact of all SAPK/MAPK is highly context dependent and influenced by interactions with regulators and scaffolding proteins, by subcellular localization and by concentration and duration of the stimulus [52]. One potential explanation of how HDACi might modulate SAPK/MAPK activity is the promotion of MAPK-phosphatase (MKP)-acetylation [53]: MKPs dephosphorylate and deactivate MAPK. Acetylation of MKP-1 enhances its activity [54,55]. In addition, transcription of MKP has been shown to be induced by activated MAPK in form of a positive feedback loop [56] and may require histone-acetylation [57]. However, the loss of treatment effect with longer reperfusion periods in this study cannot be clarified hereby.

Although with SAHA and VPA treatment we observed an increased regenerative response by Ki67 and (phospho-) YAP immunohistochemistry and (phospho-) YAP Western blot, we cannot interpret these results without bearing in mind the overall increased damage particularly in the VPA treatment group. Nonetheless, the “Hippo off” signal and dephosphorylation of YAP was detectable already with the shorter reperfusion periods of 6h and 12h, where damage seemed to be yet delayed, and which might imply a beneficial impact on regenerative capacity. An open question remains the downregulation of total YAP in HDACi treated livers early on. To elucidate the general impact of these HDACi on liver homeostasis and regeneration a partial hepatectomy (PH) model might be better suitable. For SAHA this has been investigated in a mouse model of PH with a detrimental effect of HDACi treatment on liver regeneration via blunted expression and activation of cell cycle signals downstream of cyclin D1 [58]. However, by others, an inhibiting role of HDACs in regeneration following murine PH via inhibition of cyclin D1 expression has also been demonstrated [59]. To our knowledge, the only link shown so far between HDAC inhibition and positive regulation of the Hippo-pathway by stabilization of TAZ has been established in cancer cells [60]. In the end, the role of HDACs in liver regeneration likely is complex and further investigation is warranted.

The only documented in vivo study investigating HDACi in warm hepatic I/R injury so far reported a beneficial and protective impact of Butyrate therapy [31]. It was performed on male Sprague-Dawley rats with 60min ischemia and 6-24h of reperfusion. No pre-treatment was applied. The drug was administered i.v., immediately after onset of ischemia. I/R reduced acetylation levels of H3-K9, which was partly rescued by Butyrate treatment in the early reperfusion phase (6h). Like VPA, Butyrate is a short-chain fatty acid and may similarly inhibit class I and IIa HDACs. Our opposing results may be due to the different rat strain, ischemia time, compounds used and/or treatment protocols. In another excellent, but in vitro treatment study Evankovich et al. [38] demonstrated increased cellular translocation and release of HMGB1 from murine hepatocytes treated with Trichostatin A and Scriptaid. They further elaborated a sequence in which I/R mediated HDAC inactivation and shuttling leads to increased acetylation of HMGB1 in hepatocytes with the consequence of augmented release of this classic damage associated molecular pattern and enhanced tissue damage and inflammation. Thus, adding complexity, in this model HDACs exert protective properties in hepatic I/R.

Certainly it has to be noted that this study is limited in several ways: Firstly, we selected VPA and SAHA to be able to compare two readily available and clinically approved HDACi in order to confirm the effects to be more likely deacetylase-dependent. However, HDACi are pleiotropic drugs and due to their broad spectrum, in addition to the here examined mechanisms, they do have influence on further signaling pathways like e.g. NFκB [61], may modulate autophagic flux [13,62], promote [63] or inhibit [64] angiogenesis, and are able to regulate inflammation and immunity, e.g. via modulation of TLR- and Interferon-signaling, cytokine production, antigen presentation and lymphocyte development, survival and function [65–68]. Additionally, for VPA, effects on sodium/potassium ion flux, modulation of GABAerg neurotransmitter function and further intra- or extracellular mechanisms independent of acetylation are possible [7].

Besides, isoflurane has been shown to beneficially influence intestinal I/R injury in a murine model [69]. Potential additional effects and/or interactions should be kept in mind when comparing our results with studies performed with different anesthetics.

## Conclusions

In contrast to their beneficial impact on cellular and organ viability in several settings of polytrauma, ischemia and in ischemia-reperfusion injury of the brain, kidneys and myocardium, the HDACi VPA and SAHA lack protective properties in a warm hepatic I/R rat model. Although VPA and SAHA may delay injury, at least in part via modulation of SAPK-activation, caution should be taken when considering these HDACi as (pre-) treatment options. Especially in the case of VPA belated damage is significantly aggravated.

## Supporting Information

S1 Fig VPA serum levels. Each dot represents a serum sample of one individual animal treated with VPA and subjected to I/R. Different periods (6h, 12h, 24h, 60h) of reperfusion led to different intervals from last injection to sacrifice and sample collection. Minimum threshold for HDACi effect of VPA: 0.6mmol/l (86mg/l) [3]. In vitro, histone acetylation peaks at 4h after treatment and lasts for more than 36h [34].
(TIF)

S2 Fig Phospho-YAP immunohistochemistry. Representative sections of post-ischemic and non-ischemic liver lobes from animals treated as indicated and subjected to 12h reperfusion. Loss of p-YAP staining was evident especially in the border zones to damaged areas of postischemic lobes (lower row), where nuclear YAP and ki67 staining appeared strong and frequent. In addition, loss of p-YAP staining also appeared diffusely in non-ischemic lobes, which was more pronounced in VPA and SAHA treated livers (top row). Scale bars: 100μm.
(TIF)
