# Supplementary figures and images for: HDACi Valproic Acid (VPA) and Suberoylanilide Hydroxamic Acid (SAHA) Delay but Fail to Protect against Warm Hepatic Ischemia-Reperfusion Injury
Source: PLoS One. 2016 Aug 11;11(8):e0161233. doi: 10.1371/journal.pone.0161233 (PMC4981462; doi:10.1371/journal.pone.0161233)

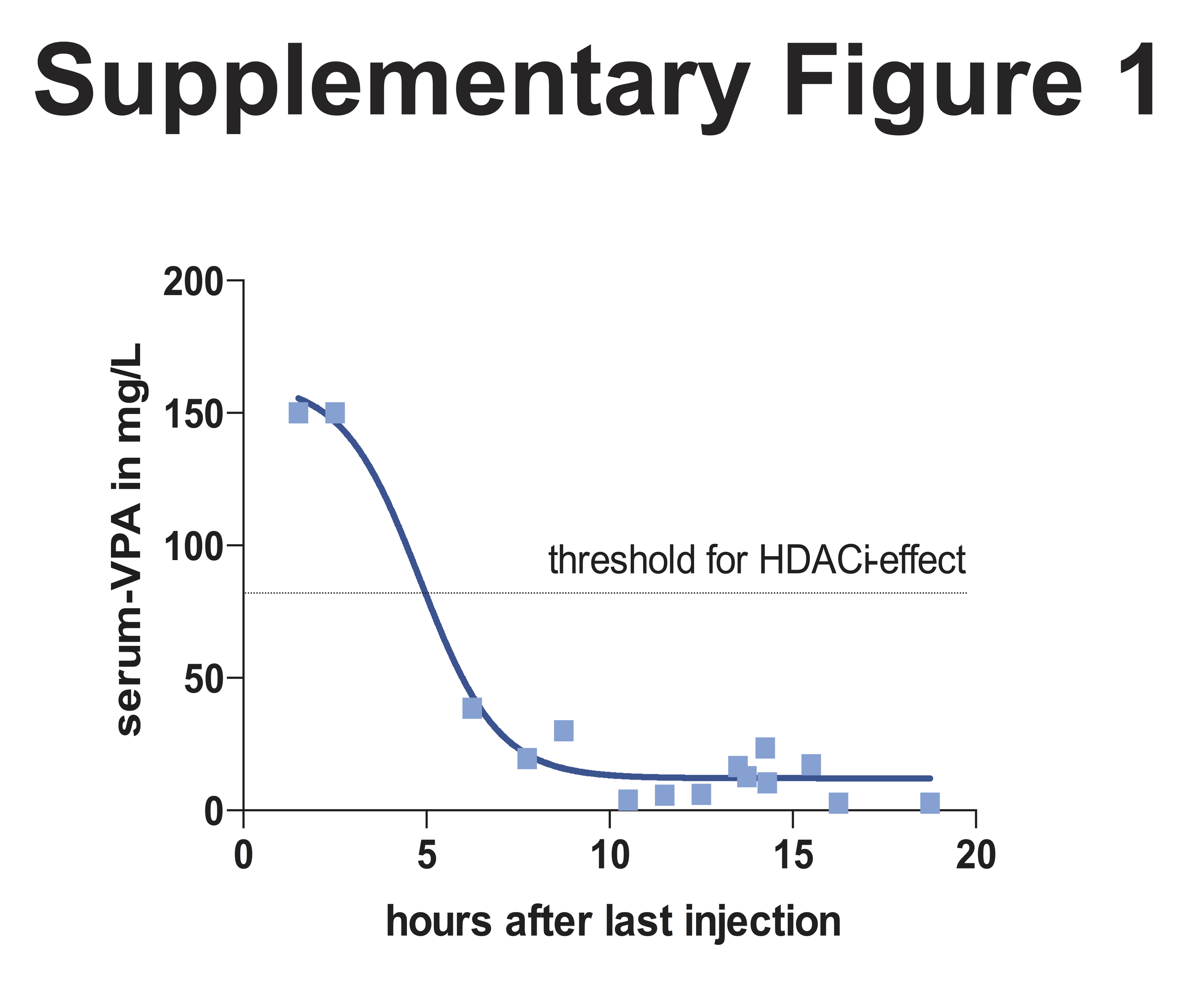

Supplement: S1 Fig — Each dot represents a serum sample of one individual animal treated with VPA and subjected to I/R. Different periods (6h, 12h, 24h, 60h) of reperfusion led to different intervals from last injection to sacrifice and sample collection. Minimum threshold for HDACi effect of VPA: 0.6mmol/l (86mg/l) [3]. In vitro, histone acetylation peaks at 4h after treatment and lasts for more than 36h [34]. (TIF) [file pone.0161233.s001.tif]

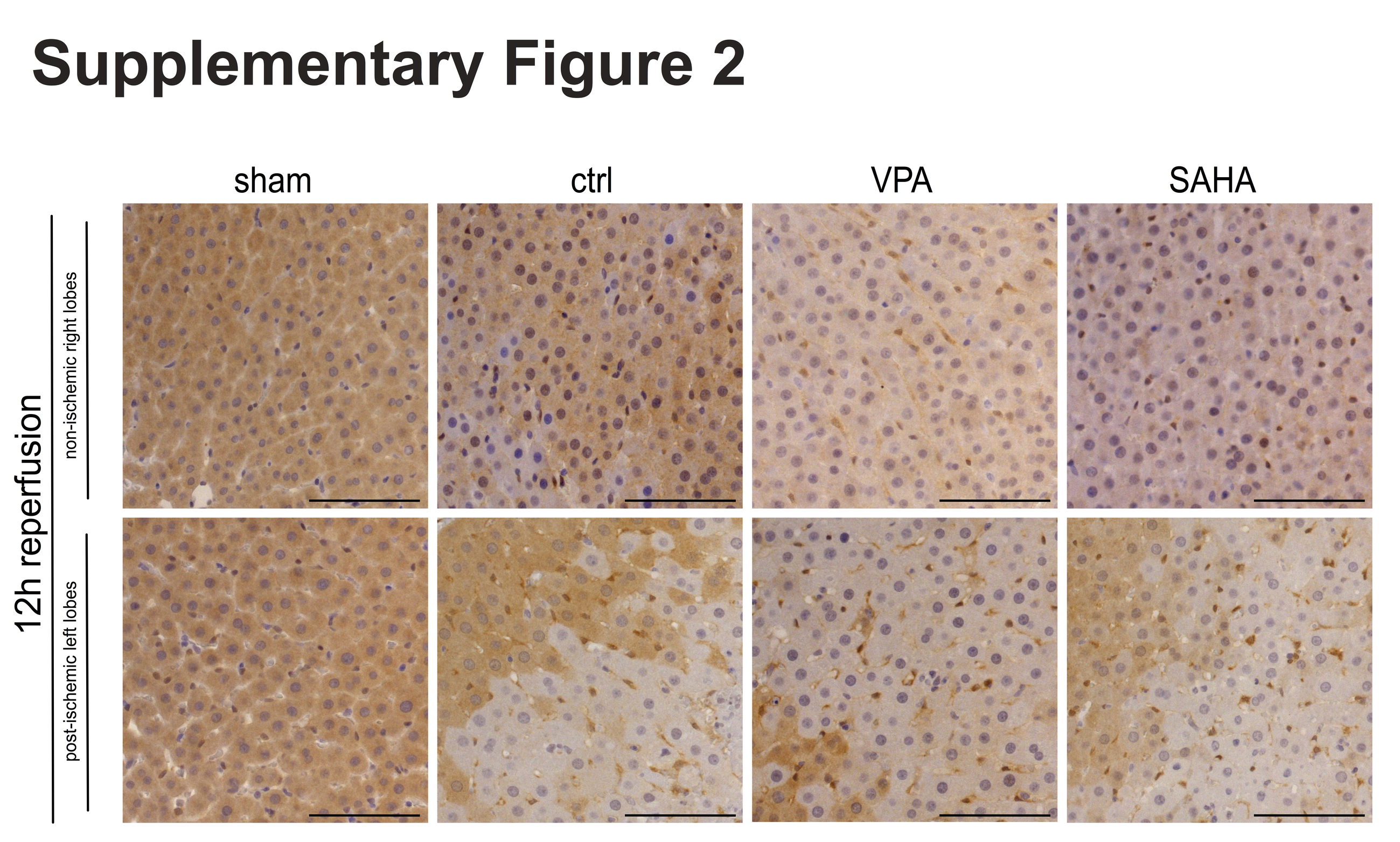

Supplement: S2 Fig — Representative sections of post-ischemic and non-ischemic liver lobes from animals treated as indicated and subjected to 12h reperfusion. Loss of p-YAP staining was evident especially in the border zones to damaged areas of postischemic lobes (lower row), where nuclear YAP and ki67 staining appeared strong and frequent. In addition, loss of p-YAP staining also appeared diffusely in non-ischemic lobes, which was more pronounced in VPA and SAHA treated livers (top row). Scale bars: 100μm. (TIF) [file pone.0161233.s002.tif]
